# High-speed video data for settling of dense liquid droplets through liquid media with different viscosities

**DOI:** 10.1016/j.dib.2020.106428

**Published:** 2020-10-20

**Authors:** Driaan Bezuidenhout, Quinn Reynolds, Markus Erwee, Oliver Oxtoby

**Affiliations:** a Mintek, South Africa; b SamancorCr, South Africa; c CSIR Aeronautic Systems, South Africa; d Engys Ltd, United Kingdom

**Keywords:** Metal-slag separation, Viscous settling, Interfacial phenomena in viscous systems, Validation of computational modelling of settling

## Abstract

A dataset of high-speed video footage of mercury droplets settling through liquid media of different viscosities is presented. Video footage was taken at 4000 frames per second for mercury droplets at room temperature settling through either deionised water or silicone oil. The data set is useful for validation of computational models of a wide range of systems which include phase separation studies, settling behaviour as well as interfacial phenomena in liquid-liquid systems. The data can serve as an analogue for validation for fluid systems where dimensional similarity exists and experimentation is not possible due to hostile experimental conditions, e.g. settling of molten metal through molten oxide slag in pyrometallurgical furnaces or separation of heavy liquid phases in chemical reactors.

## Specifications Table

**Table:** 

Subject	Computational mechanics
Specific subject area	Validation data for computational modelling of settling behaviour in multiphase liquid systems
Type of data	Tables, graphs, high-speed camera videos, photographs, STL file
How data were acquired	High-speed videography (Olympus iSpeed 3 camera with Nikon Series E 50 mm lens f/1.8)
Data format	Raw
Parameters for data collection	The settling and interaction of a dense liquid phase was captured when moving through liquids with different viscosities.
Description of data collection	Mercury droplets, at room temperature, were released into a glass beaker containing deionised water or silicone oil using an eye-dropper. The experiment was filmed using a high-speed camera to capture the settling of the mercury as it entered the water or silicone.
Data source location	Institution: Mintek City/Town/Region: Randburg Country: South Africa
Data accessibility	Repository name: High-speed video data for settling of dense liquid droplets through liquid media with different viscosities. Data identification number: DOI: 10.17632/7crhwhmvwg.2 Direct URL to data: https://data.mendeley.com/datasets/7crhwhmvwg

## Value of the Data

• The dataset can be used for validation of computational models pertaining to settling of a dense metal phase through a less dense phase of which the viscosity is either high or low.
• The dataset is particularly useful to computational scientists and chemical/metallurgical engineers studying problems of phase separation and settling (e.g. metal settling through viscous media).
• This dataset can serve as an analogue for experiments where the direct study of settling and phase separation is not possible in situ due to harsh conditions (high temperatures, high pH environments, enclosed reactors, etc.).
• Experiments were filmed with a high-speed camera, producing digital images suitable for secondary analysis. Detail of interfacial phenomena such as deformation of the interface between dense and light phase may be studied accordingly.

## 1 Data Description

In computational modelling, validation data for complex multiphase flow problems are often scarce. In the field of pyrometallurgy, which involves the study of molten metal and metal oxides processed at extreme temperatures (>1600 °C), there is even less data available to test computational models against. The liquid phases involved in pyrometallurgical processes vary widely in density, viscosity, and surface tension properties, significantly influencing flow and settling/separation phenomena.

The dimensional similarity in fluid mechanics problems offers a partial solution to studying high-temperature processes by matching their behaviour to that of materials at room temperature.

This dataset includes a set of videos taken using a high-speed camera of a dense metal phase, mercury, settling through other, less dense liquids at room temperature. In this case, the less dense liquids are silicone oils which have different viscosity values and deionised water. There are 20 raw video files – the experimental parameters (types of liquids used) for each video are listed in Table 4. An example of how the raw video data may be processed further is shown in Fig. 5.

The video files, labelled as ‘DROP0xx.hsv’, are in Olympus ‘hsv’ format. This is equivalent to Motion JPEG and can be opened by most video viewers or editors, e.g. ‘VLC media player’ [1]. For each ‘hsv’ file there is a text ‘uda’ file which can be opened using any text editor – this contains the camera settings used (constant for all videos). All “hsv.” format video files have also been converted to MPEG format for ease of use and can be found in the Mendeley data repository.

The data can serve as an analogue for validation of computational fluid dynamics models in systems where dimensional similarity exists and experimentation is not possible due to hostile experimental conditions, e.g. settling of molten metal through molten oxide slag in pyrometallurgical furnaces or separation of heavy liquid phases in chemical reactors. The high temperatures and hostile process conditions associated with the above-mentioned systems prevent the direct observation of settling behaviour, whereas computational models can offer a view inside such processes. These computational models need to be validated, which is where the data described in this paper is applied.

An adapter was 3D-printed in PLA plastic to reverse-mount the camera lens to the high-speed video camera. A standard STL file is included in the dataset as ‘Lens adapter.stl’, and may be used to reproduce a similar adapter.

## 2 Experimental Design, Materials and Methods

### 2.1 Experimental setup

**Fig. 1:**
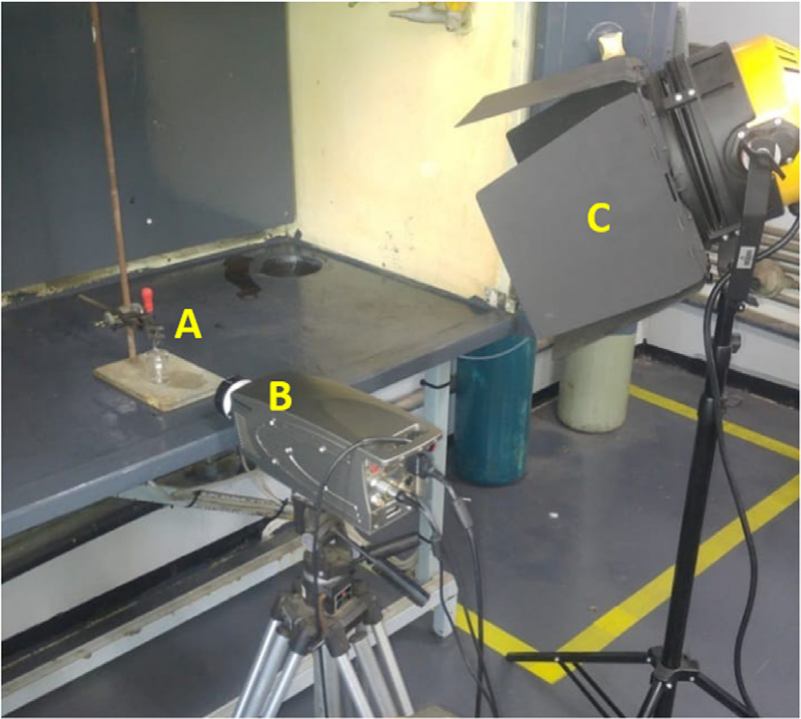
General arrangement of the experimental setup (*A* = eye-dropper fixed to retort stand over a standard borosilicate glass beaker, *B* = high-speed camera, *C* = studio light).

A standard 25 ml borosilicate glass beaker, containing some liquid mercury, was filled with silicone or deionised water and placed on the platform of a retort stand. An eye-dropper containing mercury was fixed to a clamp over the glass beaker (see ‘A’ in Fig. 1), keeping the tip of the eye-dropper submerged in the silicon or deionised water (‘light –phase’).

An Olympus iSpeed 3, full-colour 4GB model with a Nikon Series E 50 mm f/1.8 lens was used to capture the high-speed video (‘B’ in Fig. 1). Lighting was provided using a studio light fixture containing a 2000 W, 240 V, halogen optic lamp manufactured by OSRAM (Pty.) Ltd. (‘C’ in Fig. 1).

**Fig. 2:**
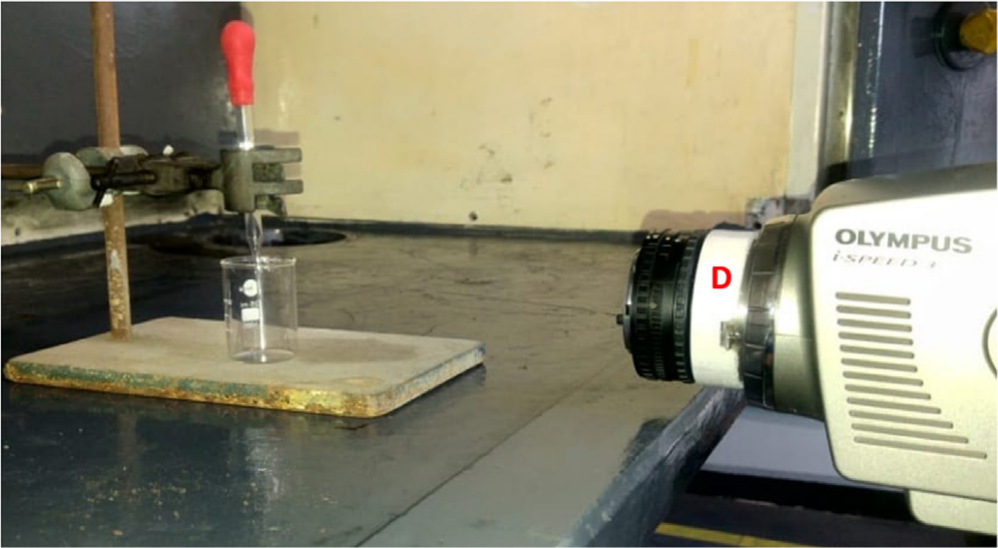
Detail arrangement of camera and eye-dropper (*D* = 3D-printed lens adapter to connect the reversed camera lens to the high-speed camera).

The lens was mounted to the camera in reverse using the 3D-printed adapter to provide additional magnification of the experimental area of interest (see ‘D’ in Fig. 2). White balance correction was performed using the camera's automatic adjustment function during the initial setup of the experimental equipment and remained unchanged for all tests. Recording and clip selection were performed manually by the camera operator during and after each test.

Once all of the components were in place, the studio light was switched on and the mercury droplet released by squeezing the rubber on the eye-dropper. Mercury droplets were released in either silicon oil or deionized water as detailed in section 0 of this paper. The high-speed video camera recording cycle was activated as the mercury droplet was released, allowing a video clip to be selected illustrating the mercury droplet decent, through the ‘light-phase’, and contact with the mercury bath.

**Fig. 3:**
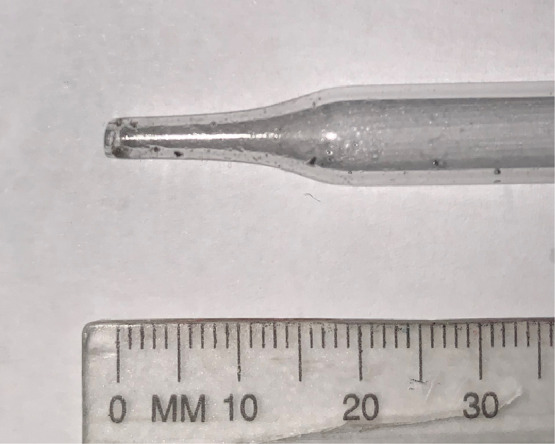
Micro still photograph of the dropper tip.

**Fig. 4:**
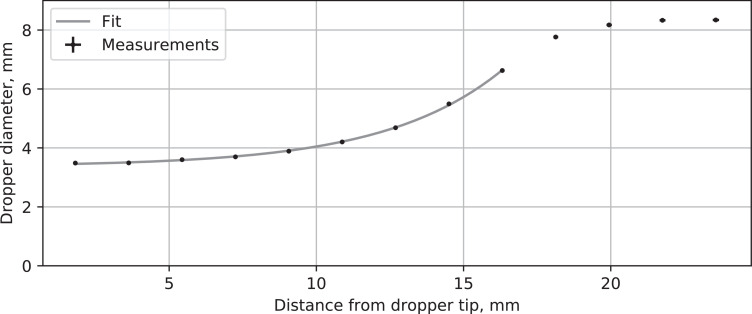
Measured diameter of dropper at different distances from the tip (cross sizes show estimated measurement error).

The eye-dropper used to release the mercury droplets is the only available scale reference in the video image data. The eye-dropper's dimensions were quantified by taking a macro still photograph (Fig. 3) and measuring the dropper diameter at different distances from the tip using a Vernier caliper. Measurements were repeated 5 times, with the minimum and maximum readings used to estimate the error. A similar procedure was followed for a calibration ruler included in the image. The results with combined measurement errors are shown in Table 1 and Fig. 4. The dropper dimensions for the tip section were also fitted to an empirical equation for easier analysis:

d=3.371+0.05554exp(0.2498z)

where dis the dropper diameter and zis the distance from the tip, both in mm.

**Table 1:** Measured diameter of dropper at different distances from the tip.

z: avg (min, max) [mm]	d: avg (min, max) [mm]
1.813 (1.805, 1.821)	3.486 (3.466, 3.506)
3.626 (3.610, 3.643)	3.492 (3.466, 3.515)
5.439 (5.415, 5.464)	3.601 (3.565, 3.634)
7.252 (7.220, 7.286)	3.697 (3.673, 3.725)
9.065 (9.025, 9.107)	3.889 (3.863, 3.916)
10.878 (10.830, 10.929)	4.202 (4.170, 4.244)
12.691 (12.635, 12.750)	4.685 (4.648, 4.727)
14.504 (14.440, 14.572)	5.493 (5.451, 5.546)
16.316 (16.245, 16.393)	6.626 (6.588, 6.667)
18.129 (18.051, 18.215)	7.763 (7.717, 7.814)
19.942 (19.856, 20.036)	8.173 (8.123, 8.224)
21.755 (21.661, 21.858)	8.330 (8.285, 8.379)
23.568 (23.466, 23.679)	8.341 (8.294, 8.397)

### 2.2 Materials

The liquid materials used were deionised water, triple-distilled metallic mercury (Minema chemicals, CAS No 7439–97–6) [2] and silicone oil viscosity standards (Brookfield Ametek) [3]. The densities of the deionised water, silicone oil, and mercury are listed in Table 2 at 25 °C. The silicone oil standards are typically used for calibration of viscometers, and their viscosities are quoted to be accurate within 1% by the manufacturer. Two silicone oils were used, with viscosities of 0.0966 Pa.s and 0.498 Pa.s at 25 °C. These two viscosity standards were selected as it cover a range of possible liquid slag viscosities, which is what this data was intended for.

**Table 2:** Material properties of liquid materials used during the experiment.

Material	Viscosity [Pa.s] @ 25 °C	Density [kg/m^3^] @ 25 °C
Deionised water	0.00548 [4]	982.3 [4]
100 cP Viscosity standard	0.0966 [3]	965 [3]
500 cP Viscosity standard	0.498 [3]	970 [3]
Triple-distilled metallic mercury	0.001544 [4]	13,550 [2]

### 2.3 Experimental conditions

The experimental conditions are given in Table 3. The ‘dense phase’ refers to mercury settling through the ‘light phase’ which is either silicone oil or water. Note that tests 2 to 11 were repeats of test 1 (mercury-oil with oil viscosity of 0.0966 Pa.s), while tests 13 and 14 are repeats of test 12 (mercury-oil with oil viscosity of 0.498 Pa.s). Tests 16 to 20 are a repeat of test 15 (mercury-water).

**Table 3:** Test conditions for settling experiments.

Test number	Dense phase	Light phase	Video file
1	Mercury	Silicone oil, 0.0966 Pa.s	DROP002.hsv
2	Mercury	Silicone oil, 0.0966 Pa.s	DROP003.hsv
3	Mercury	Silicone oil, 0.0966 Pa.s	DROP004.hsv
4	Mercury	Silicone oil, 0.0966 Pa.s	DROP005.hsv
5	Mercury	Silicone oil, 0.0966 Pa.s	DROP006.hsv
6	Mercury	Silicone oil, 0.0966 Pa.s	DROP007.hsv
7	Mercury	Silicone oil, 0.0966 Pa.s	DROP008.hsv
8	Mercury	Silicone oil, 0.0966 Pa.s	DROP012.hsv
9	Mercury	Silicone oil, 0.0966 Pa.s	DROP013.hsv
10	Mercury	Silicone oil, 0.0966 Pa.s	DROP014.hsv
11	Mercury	Silicone oil, 0.0966 Pa.s	DROP015.hsv
12	Mercury	Silicone oil, 0.498 Pa.s	DROP016.hsv
13	Mercury	Silicone oil, 0.498 Pa.s	DROP017.hsv
14	Mercury	Silicone oil, 0.498 Pa.s	DROP018.hsv
15	Mercury	Deionised water	DROP019.hsv
16	Mercury	Deionised water	DROP020.hsv
17	Mercury	Deionised water	DROP021.hsv
18	Mercury	Deionised water	DROP022.hsv
19	Mercury	Deionised water	DROP023.hsv
20	Mercury	Deionised water	DROP024.hsv

### 2.4 Settings and parameters used for high-speed camera

Settings used for the camera are shown in Table 4.

**Table 4:** Camera settings for video recording.

Parameter	Setting
Recording rate	4000 frames per second
Frame-to-frame time	0.25 ms
Shutter speed	0.25 ms
Trigger	Manual (falling edge)
Video resolution	608 × 688
Video file format	Olympus HSV (MJPEG, 7:1 compression)

### 2.5 Example of image correction and analysis

**Fig. 5:**
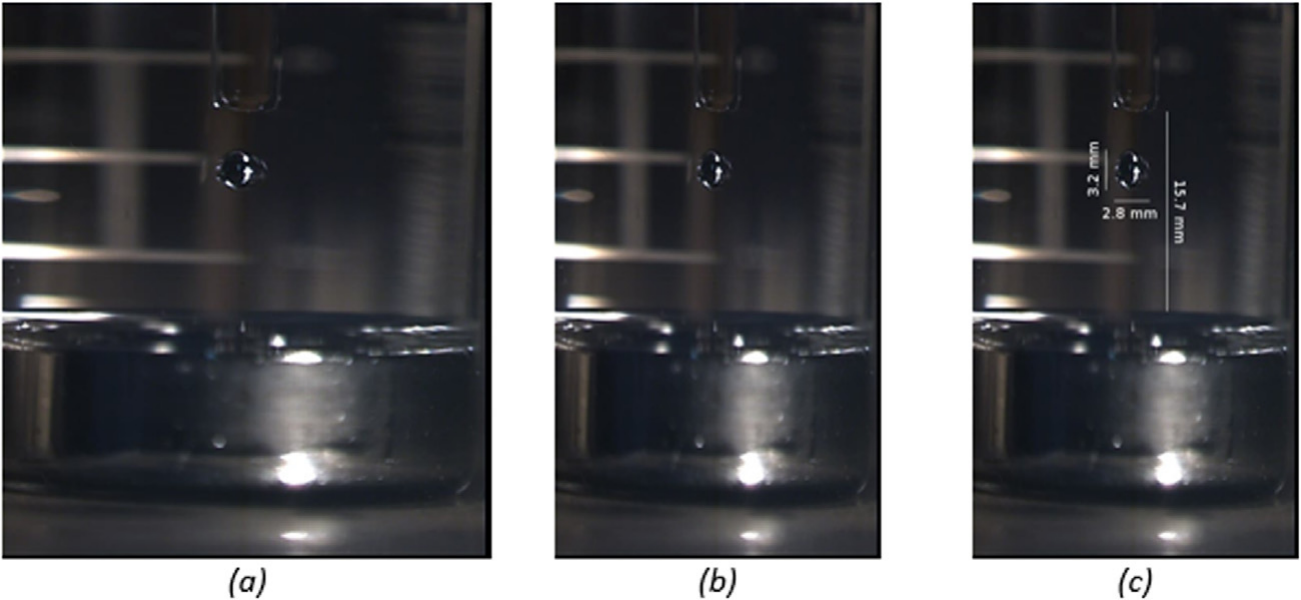
Example of basic image analysis for frame 4 of 456 from test 3 (DROP004), (a) raw image, (b) correction for distortion, (c) annotated with measurements.

The experimental setup involves photography of the droplet impact through a cylindrical glass beaker in air. Assuming dropper and droplet are positioned on the beaker's centreline, the system behaves optically as an aplanatic semi-circular lens and magnifies the region of interest in the horizontal direction by a factor of $n_{b}/n_{a}$, where $n_{b}$and $n_{a}$are the refractive indices of the beaker material and air respectively [5]. In the case of the borosilicate glass beakers used in this experiment, the magnification factor is approximately 1.51 [6], and the raw frame images from the video files must be resized accordingly to compensate for the distortion of the image. This is shown in Fig. 5b.

After distortion correction, pixel-to-length conversion information may be obtained with manual or automated image processing methods using the shape of the dropper tip as a reference length. In this example case the diameter of the dropper tip in pixels was measured at several locations using ImageJ 1.52p [7], and relevant dimensions of interest measured and converted to mm using the dropper tip shape information given in Fig. 4. The results are shown in Fig. 5c.

## 3 Safety, health, environment, and quality requirements

All procedures were followed in accordance with the material safety data sheets and comply with all regulations, legislation, conventions and requirements that affect Safety, Health, Environment, Quality and Radiation Protection.

The MSDS for the triple distilled mercury from Minema chemicals (CAS No 7439–97–6) can be found at the following link - http://www.minema.co.za/MSDSpdf/M5100

The MSDS for the 0.0966 Pa.s silicone oil viscosity standards by Brookfield Ametek can be found at the following link -https://www.brookfieldengineering.com/-/media/ametekbrookfield/safety%20data%20sheets/silicone%20viscosity%20standards%20general%20purpose/sds%20silicone%20100%20cp.pdf?la=en

The MSDS for the 0.498 Pa.s silicone oil viscosity standards by Brookfield Ametek can be found at the following link -https://www.brookfieldengineering.com/-/media/ametekbrookfield/safety%20data%20sheets/silicone%20viscosity%20standards%20general%20purpose/sds%20silicone%20500%20cp.pdf?la=en

The MSDS for deionised water can be found at the following link -https://www.fishersci.com/shop/msdsproxy?storeId=10652&productName=23751628

## Declaration of Competing Interest

The authors declare that they have no known competing financial interests or personal relationships which have, or could be perceived to have, influenced the work reported in this article.
